# Anti-apoptotic effects of IGF-I on mortality and dysmorphogenesis in *tbx5*-deficient zebrafish embryos

**DOI:** 10.1186/s12861-017-0161-1

**Published:** 2018-03-05

**Authors:** Tzu-Chun Tsai, Chun-Che Shih, Hsin-Ping Chien, An-Hang Yang, Jenn-Kan Lu, Jen-Her Lu

**Affiliations:** 1 Institutes of Clinical Medicine, School of Medicine, National Yang-Ming University, Taipei, Taiwan, Republic of China; 2 Departments of Surgery, Pediatrics and Pathology, Veterans General Hospital-Taipei, Taipei, Taiwan, Republic of China; 3 Laboratory of Molecular Biology, Institute of Aquaculture, National Taiwan Ocean University, Keelung, Taiwan, Republic of China; 4 Department of Pediatrics, Veterans General Hospital-Taipei, No. 201, Shih-Pei Rd., Section 2, Beitou, Taipei, 112 Taiwan, Republic of China

**Keywords:** Apoptosis, Holt-Oram syndrome, Insulin-like growth factor I, tbx5, Zebrafish

## Abstract

**Background:**

*Tbx5* deficiency in zebrafish causes several abnormal phenotypes of the heart and pectoral fins. It has been reported that exogenous human growth hormone can enhance expression of downstream mediators in the growth hormone and insulin-like growth factor I (IGF-I) pathway and partially restore dysmorphogenesis in *tbx5* morphants. This study aimed to further evaluate the effects of IGF-I on cell apoptosis and dysmorphogenesis in zebrafish embryos deficient for *tbx5*.

**Results:**

Among the five studied groups of zebrafish embryos (wild-type embryos [WT], *tbx5* morphants [MO], mismatched *tbx5* morpholino-treated wild-type embryos [MIS], IGF-I-treated wild-type embryos [WTIGF1], and IGF-I-treated *tbx5* morphants [MOIGF1]), the expression levels of the *ifg1*, *igf1-ra, ifg-rb, erk1,* and *akt2* genes as well as the ERK and AKT proteins were significantly reduced in the MO group, but were partially restored in the MOIGF1 group. These expression levels remained normal in the WT, MIS, and WTIGF1 groups. Exogenous human IGF-I also reduced the incidence of phenotypic anomalies, decreased the expression levels of apoptotic genes and proteins, suppressed cell apoptosis, and improved survival of the MOIGF1 group.

**Conclusions:**

These results suggest that IGF-I has an anti-apoptotic protective effect in zebrafish embryos with *tbx5* deficiency.

## Background

*tbx5* mutations in humans cause autosomal-dominant Holt-Oram syndrome, which is characterized by congenital cardiac and forearm anomalies. Although the types or locations of mutations cannot predict the severity of the malformation, a consistent heart or limb/pectoral fin phenotype exists in different species with *tbx5* deficiency [1–7]. Deficiency in *tbx5* alters multiple transcriptional expression patterns, interferes with mesoderm migration, and causes a wide spectrum of cardiac defects, along with hypogenesis or complete agenesis of the pectoral fins and shortened curved trunks in zebrafish [8, 9]. The malformations caused by *tbx5* deficiency have previously been described as “premature stops” or “developmental delay” during embryogenesis [10]. The mechanism of abnormal embryogenesis caused by *tbx5* deficiency remains unclear. However, previous experiments have suggested that aberrant apoptosis is a key event in TBX5 deficiency-related dysmorphogenesis [11]. Knockdown of the *tbx5* gene in zebrafish embryos induces apoptosis and inhibits cell growth, which contribute to cardiac and forearm defects. Furthermore, excessive aberrant apoptosis in hearts and pectoral fins coincides with developmental “stops” on the fail-to-looping heart and fail-to-outgrowing fins [10, 11]. *tbx5* deficiency also diminishes the expression levels of *amhc*, *vmhc*, and *cmlc2* as well as disturbs cardiomyogenesis, contributing to string-heart phenotypes in zebrafish embryos [12].

The *tbx5* gene is a TBX5 transcriptional factor and a member of the T-box gene family located on chromosome 12q24 in human and chromosome 5 in zebrafish. The similarity of the amino acid sequences of TBX5 between human and zebrafish is greater than 95% [3, 6]. TBX5 is expressed on the pectoral fin bud mesenchyme at the 18-somite stage and on ring-like cardiac precursors at the 20-somite stage and involves migration of precursor mesenchymes to form the fins and heart [1, 8, 9]. TBX5 harbors multiple distinct conserved transcriptional regulatory domains, accounting for its roles in successful formation of the heart and limbs/fins [6].

Insulin-like growth factor I (IGF-I) acts in an exocrine, paracrine and also endocrine manner during embryogenesis in zebrafish and other fishes [13]. Binding of IGF-I to its receptor activates several downstream signaling cascades, including the AKT and MAP kinase pathways, which are highly conserved in fishes and mammals [14, 15]. Activation of AKT signaling stimulates a phosphorylation cascade that provokes sequential translation and protein syntheses [14, 15]. IGF-I plays an important role during skeletal development by promoting chondrocyte proliferation and maturation as well as inhibiting apoptosis to achieve appropriate skeletal mass and strength [16]. IGF-I is also viewed as a cardiac hormone since the IGF-I pathway promotes the development of cardiomyocytes and improves cardiac function by directly acting on cardiomyocytes [17]. Blockade of IGF-I-related pathways directly influences normal cardiac development and induces cardiac malformation and dysfunction [18–20].

We have previously observed that exogenous human growth hormone (GH) treatment can enhance expression of downstream mediators in the GH and IGF-I pathways and partially restore dysmorphogenesis in *tbx5* morphants [21]. Therefore, this study aimed to further evaluate the effects of IGF-I on cell apoptosis and dysmorphogenesis in *tbx5*-deficient zebrafish embryos by microinjecting exogenous recombinant human IGF-I into *tbx5*-deficient zebrafish embryos at an extremely early embryonic stage.

## Methods

### Animal ethics

Approval of this experiment was given by the Animal Ethics Review Board of National Taiwan Ocean University Aquaculture. Since zebrafish embryos are not considered to be “vertebrate animals” until 7 days (168 h) post-fertilization, the zebrafish embryos used in this study were less than 48 h post-fertilization (hpf) and were not subject to the regulation and review process of the Basic Institutional Review Board.

### Maintenance of zebrafish

Zebrafishes were maintained in 45-l aquaria heated to 28.5 °C with 25 fishes per tank. The water was filtered, and approximately half of the water was replaced at least once per week. Adult zebrafishes were fed once to twice daily with a variety of food, and young zebrafishes were fed twice daily [7]. The tank was cleaned daily by siphoning off any excess food after the second daily feeding. The day-night cycle was controlled with an automatic timer (14 h light, and 10 h dark).

### Zebrafish breeding and embryo collection

Zebrafishes reach sexual maturity within 10–12 weeks, but breeding fish should be between 7 and 18 months of age for maximum embryonic production. The day before breeding, 1/3 of the water was replaced and the tank was cleaned after feeding (1–2 h before the end of the light period). A collection box was placed at the bottom of the tank, and preparations were made to collect the embryos the next day. When the light was turned on, we removed the collection box and placed the collected embryos into an incubator maintained at 28.5 °C.

### Construction of zebrafish embryos with different genotypes and treatment

This study included five groups: wild-type embryos (WT), *tbx5* morphants (MO), mismatched *tbx5* morpholino-treated wild-type embryos (MIS), IGF-I-treated wild-type embryos (WTIGF1), and IGF-I-treated *tbx5* morphants (MOIGF1). Microinjection was used for the construction and treatments.

MO group (*tbx5* morphants): A morpholino antisense oligonucleotide targeting *tbx5* (5-GAAAGGTGTCTTCACTGTCCGCCAT-3) was designed to block the *tbx5* translational start site (Gene Tools LLC, Philomath, OR, USA). A 19.4 ng/4.6 nl stock morpholino diluted in Danieau’s solution was microinjected into wild-type embryos that were primarily at the 1-cell stage with intact chorions.

MIS group (mismatched *tbx5* morpholino-treated wild-type embryos): 1-cell stage wild-type embryos (*n* = 150/group, in triplicate) were microinjected with mismatched *tbx5* morpholino (5’-GTCTCTTGACTCTCCGCGATCTCGG-3′) [11, 12].

WTIGF1 group (IGF-I-treated wild-type embryos): 1-cell stage wild-type embryos (n = 150/group, in triplicate) were microinjected with 1 pmol/2.3 nl human IGF-I (Sigma-Aldrich, St. Louis, MO, USA).

MOIGF1 group (IGF-I-treated *tbx5* morphants): 1-cell stage wild-type embryos (n = 150/group, in triplicate) were microinjected with 1 pmol/2.3 nl human IGF-I and 19.4 ng/2.3 nl of *tbx5* morpholino.

Our previous studies used four control groups, including the 3′ end of the *tbx5* morpholino (2) (5’-GCCTGTACGATGTCTACCGTGAGGC-3′), mismatched *tbx5* morpholino (5’-GTCTCTTGACTCTCCGCGATCTCGG-3′), wild-type embryos that received a blank microinjection, and wild-type embryos that did not received a microinjection to identify the specific inhibitory effects on *tbx5* mRNA translation by the *tbx5* morpholino. The effectiveness and specificity of the *tbx5* morpholino has been verified by western blot analysis for green fluorescent protein [11, 12].

### RNA isolation

Total RNA was isolated from 50 embryos using the guanidine isothiocyanate-based TRIzol solution (Life Technologies, UK). RNA samples were resuspended in diethylpyrocarbonate-treated water and quantified spectrophotometrically at 260 nm. The RNA quality was then checked by 1.2% agarose gel electrophoresis after staining with 1 μg/ml ethidium bromide. The RNA stock was stored at −80 °C.

### Microarray analysis

RNA from WT, MO, and MOIGF1 embryos at 24 hpf, 30 hpf, and 48 hpf were isolated and purified by using a RNeasy® Mini Kit (QIAGEN, Hilden, Germany), and the RNA quality was confirmed using an Agilent 2100 Bioanalyzer (Agilent Technologies, Santa Cruz, CA, USA). Purified RNA was reverse-transcribed into cDNA by using SuperScript TM III RT (Invitrogen, Carlsbad, CA, USA). Before purifying and coupling with a fluorescent dye with indirect cDNA labeling for analysis via a zebrafish-specific microarray kit of 43,663 transcripts (Invitrogen, Carlsbad, CA, USA), cDNA was hydrolyzed and neutralized using NaOH and HCl. cDNA was then pretreated with GEx hybridization buffer HI-PRM (Agilent Technologies, Santa Cruz, CA, USA) before transferring to hybridization chamber gasket slides for hybridization. The slides were scanned by a GenePix 4000B Axon Instruments scanner (Molecular Devices, Silicon Valley, CA, USA), and data were analyzed by using a Genespring GX 10.0.2, Agilent (Agilent Technologies, Santa Cruz, CA, USA).

Microarray analyses were performed for the WT, MO, and MOIGF1 groups under the same conditions to understand the effects of IGF-I on *tbx5* morphants. The expression of a certain gene in the WT group was used as a reference and set to a value of 1. If the expression levels of the same gene in the MO group and MOIGF1 group were both more than 1.5-fold higher than that in the WT group, the gene was marked as “up-regulated”. Conversely, if the expression levels of the same gene in the MO group and MOIGF1 group were both more than 1.5-fold lower than that in the WT group, the gene was marked as “down-regulated”. These comparisons were analyzed at 24 hpf, 30 hpf, and 48 hpf. All gene transcripts (43663) were screened at these three timepoints. All data were compliant with Minimum Information about a Microarray Experiment, and the raw data were deposited in a Gene Expression Omnibus database (GSE33965) [NCBI tracking system #16217606].

### Semi-quantitative real-time reverse-transcription polymerase chain reaction (RT-PCR)

Total RNA was prepared from 50 defective or normal embryos (Invitrogen Corporation, Carlsbad, CA, USA). Three microliters of 1st-strand cDNA was amplified. The amplification primers for each specific mRNA were deduced from published sequences: *igf1* (P1: 5’-TCTCATCCTCTTTCTCGC-3′, P2: 5’-GATAGTTTCTGCCCCC-3′), *igf1ra* (P1: 5’-CAAGCCTGGATAAACAC-3′, P2: 5’-GGCAGATTGAAAGAAAG-3′), *igf1rb* (P1: 5’-AAGCATTGAGAGGTG-3′, P2: 5′-AGAGGAAGTGAGGAGAA-3′), *akt2* (P1: 5’-GAAGAGGATGAGCCAATG-3′, P2: 5’-CTCCAACGCTGAAACAAT-3′), *erk1* (P1: 5’-TCTGCCAATGTGCTGC-3′, P2: 5’-TGCCGTCTCCTCAAAG-3′). The PCR conditions were as follows: denaturation at 95 °C for 3 min, followed by 50 cycles of amplification (95 °C for 20 s, 59 °C for 15 s, and 72 °C for 20 s). The reference gene was β-actin. The gene expression levels are presented as the relative expression, which was defined as gene expression/β-actin expression.

### Western blot analysis

Embryos at 48 hpf were homogenized on ice in lysis buffer (Sigma-Aldrich, St. Louis, MO, USA). Then, cellular debris was pelleted by centrifugation at 12,000 rpm for 20 min, and the supernatant was collected and measured. Proteins were mixed with sample buffer before separating via 10% sodium dodecylsulfate polyacrylamide gel electrophoresis and were then transferred onto nitrocellulose membranes at 100 V for 1 h. Membranes were blocked with blocking buffer (5% bovine serum albumin) at room temperature for 1 h. The AKT and ERK primary antibodies (Aviva Systems Biology LLC, San Diego, CA, USA) were incubated overnight at 4 °C using the appropriate dilution (1:1000). The nitrocellulose membranes were washed with phosphate-buffered saline, followed by incubation with a horseradish peroxidase-conjugated secondary antibody (1:5000) for 1 h at room temperature before the images were acquired.

### Immunohistochemical analysis

Zebrafish embryos were fixed with 4% paraformaldehyde in phosphate-buffered saline. Deparaffinized sections (3 μm-thick) of zebrafish embryo tissues were placed on slides and processed for immunohistochemistry. After application of a biotin blocking system (Dako, Glostrup, Denmark) for 30 min, sections were incubated with target-purified rabbit primary antibodies, including MF20, Cx43, MEF, BAD, and BCL2 (all from Anaspec, Fremont, CA, USA); washed in phosphate-buffered saline; and then incubated with a rhodamine-conjugated secondary antibody, goat anti-rabbit immunoglobulin G (IgG). After washing in phosphate-buffered saline, sections were incubated with mounting medium and kept at 4 °C.

### Terminal deoxynucleotidyl transferase 2′-deoxyuridine 5′-triphosphate nick end labeling assay (TUNEL)

Both whole mount and sectioned TUNEL assays were performed using an ApopTag kit (Chemicon, Heule, Belgium). Briefly, zebrafish embryos were fixed with 4% paraformaldehyde in phosphate-buffered saline. Proteinase K-treated whole embryos or deparaffinized sections (5 μm-thick) of embryos were incubated with the terminal deoxynucleotidyl transferase enzyme followed by anti-digoxigenin. Finally, embryos or slides were stained with diaminobenzidine for 5 min.

### Survival rate assessment

Zebrafish embryos after injection were incubated in 6-well plates for observation at 12- to 18-h intervals. The survival rate was evaluated every 12 h until 84 hpf.

### Statistical analysis

The results are presented as the means ± standard deviation. Duncan’s new multiple range testing was used to compare every pair of test groups. Statistical significance was accepted at *p* < 0.05.

## Results

### Microarray analysis of the tbx5 morphants

Using a microarray approach, we compared the RNA expression levels in whole embryos between the WT, MO, and MOIGF-1 groups at 24, 30 and 48 hpf. The results are presented in Table 1. After screening all of the genes, it was noted that most of the IGF-I-related genes were down-regulated during the three stages of organogenesis (24 hpf, 30 hpf, and 48 hpf). The majority of up-regulated genes were ribosomal protein genes. Several apoptosis-related genes and phosphorylation-related genes in the IGF-I pathway were also either up-regulated or down-regulated during the process of organogenesis (Table 1).

**Table 1:** Results of microarray analysis of *tbx5 knockdown zebrafish embryos*

GenBank Accession	Gene Symbol	Gene Name	Biological Process	Stages	Reference
Up-regulated genes ^a^					
NM_131188	*mylz2*	myosin, light polypeptide 2, skeletal muscle	phosphorylation	24	Xu Y, et al. 2010
NM_178436	*mcm5*	MCM5 minichromosome maintenance deficient 5 (*S. cerevisiae*)	cell cycle, apoptosis	30	Soojin Ryu, et al. 2005
NM_130925	*khdrbs1*	KH domain containing, RNA binding, signal transduction associated 1	cell cycle, phosphorylation	24,30	Paronetto MP, et al. 2011
NM_130926	*nme2*	non-metastatic cells 2, protein (NM23B) expressed in	cell cycle, phosphorylation	30,48	Prakash T, et al. 2010
NM_131404	*pcna*	proliferating cell nuclear antigen	cell cycle	24,30,48	Sapna D B, et al. 2010
NM_200650	*prmt1*	protein arginine methyltransferase 1	cell cycle, apoptosis	24,30,48	A. Scoumanne, et al. 2009
*S ribosomal proteins*					
NM_201153	*rps3*	ribosomal protein S3	cell cycle, apoptosis	24,30,48	Sang Bae Lee, et al. 2010
NM_214793	*rps8*	ribosomal protein S8	apoptosis	30	Hao Y, et al. 2011
NM_200750	*rps19*	ribosomal protein S19	cell cycle, apoptosis,	24,30,48	K. Miyake, et al.2008
*L ribosomal proteins*					
NM_001003844	*rpl6*	ribosomal protein L6	cell cycle	30,48	Wu Q, Gou Y, Wang Q, Jin H, Cui L, et al. 2011
NM_213644	*rpl7*	ribosomal protein L7	cardiac myogenesis, cell cycle, apoptosis	24,30,48	Neumann F, et al. 1995
NM_212784	*rpl13a*	ribosomal protein L13a	apoptosis	24,30,48	Hu, B., et al. 2009
NM_213107	*rpl4*	ribosomal protein L4	cardiac myogenesis, apoptosis	24,30,48	Zhu, H, et al. 2007
Down-regulated genes ^a^					
NM_001001832	*ift57*	intraflagellar transport protein 57	apoptosis	30,48	Sarmah B.*,* et al. 2007
NM_001020710	*pskh1*	protein serine kinase H1	phosphorylation	30,48	Brede G., et al. 2003
NM_001003738	*ciapin1*	cytokine induced apoptosis inhibitor 1	cell cycle, apoptosis	30,48	Li X, Hao Z, Fan R*,* et al. 2007
NM_001077567	*eif2ak1*	eukaryotic translation initiation factor 2-alpha kinase 1	phosphorylation	30,48	Rothenburg S, et al. 2005
NM_001013443	*eif4g2b*	eukaryotic translation initiation factor 4, gamma 2b	apoptosis	30,48	Nousch, M., Reed, V., et al. 2007
NM_001030253	*bcl2*	B-cell leukemia/lymphoma 2	apoptosis	30,48	Kratz, E., et al. 2006
NM_131837	*cebp1*	CCAAT/enhancer binding protein (C/EBP) 1	cell cycle	30,48	Kitaguchi, T., et al. 2009
NM_199784	*eapp*	e2f-associated phosphoprotein	apoptosis	30,48	Novy M.*,* et al. 2005
NM_001102387	*nox1*	NADPH oxidase 1	cardiac myogenesis	30,48	Jian Li, et al. 2006
NM_182885	*pcdh10b*	protocadherin 10b	cell cycle	30,48	Murakami, T., et al. 2006
NM_200348	*cdc73*	cell division cycle 73, Paf1/RNA polymerase II complex component, homolog (S. cerevisiae)	cardiac myogenesis, cell cycle	30,48	Bai, X., et al. 2004
NM_173236	*hdac1*	histone deacetylase 1	cell cycle, apoptosis	24,48	Burns, C.E., et al. 2009
NM_199481	*ccng1*	cyclin G1	cell cycle	24,48	Newman, M., et al. 2009
NM_001002198	*btbd9*	BTB (POZ) domain containing 9	cell cycle	24,30,48	Schormair B, et al. 2011
NM_001079826	*bida*	BH3 interacting domain death agonist	apoptosis	24,30,48	Kratz, E*.,* et al. 2006
NM_001039634	*wt1b*	Wilms tumor 1b	apoptosis	24,30,48	Perner B, et al. 2007
NM_198146	*akt2*	v-akt murine thymoma viral oncogene homolog 2	apoptosis, phosphorylation	24,30,48	Jensen, P.J., et al. 2010
NM_131390	*gsk3a*	glycogen synthase kinase 3 alpha	cardiac mophogenesis	24,30,48	Lee, H.C., et al. 2007

^a^The expression of genes in the WT group was taken as the reference of 1. If the expression of a gene in the MO group and in the MOIGF1 group were both higher than 1.5 folds of that in the WT group, the gene would be marked as “up-regulation”. Conversely, if the expression of a gene in the MO group and in the MOIGF1 group were both lower than 1.5 folds of that in the WT group, the gene would be marked as “down-regulation”

### Phenotypes of the tbx5 morphants

**Fig. 1:**
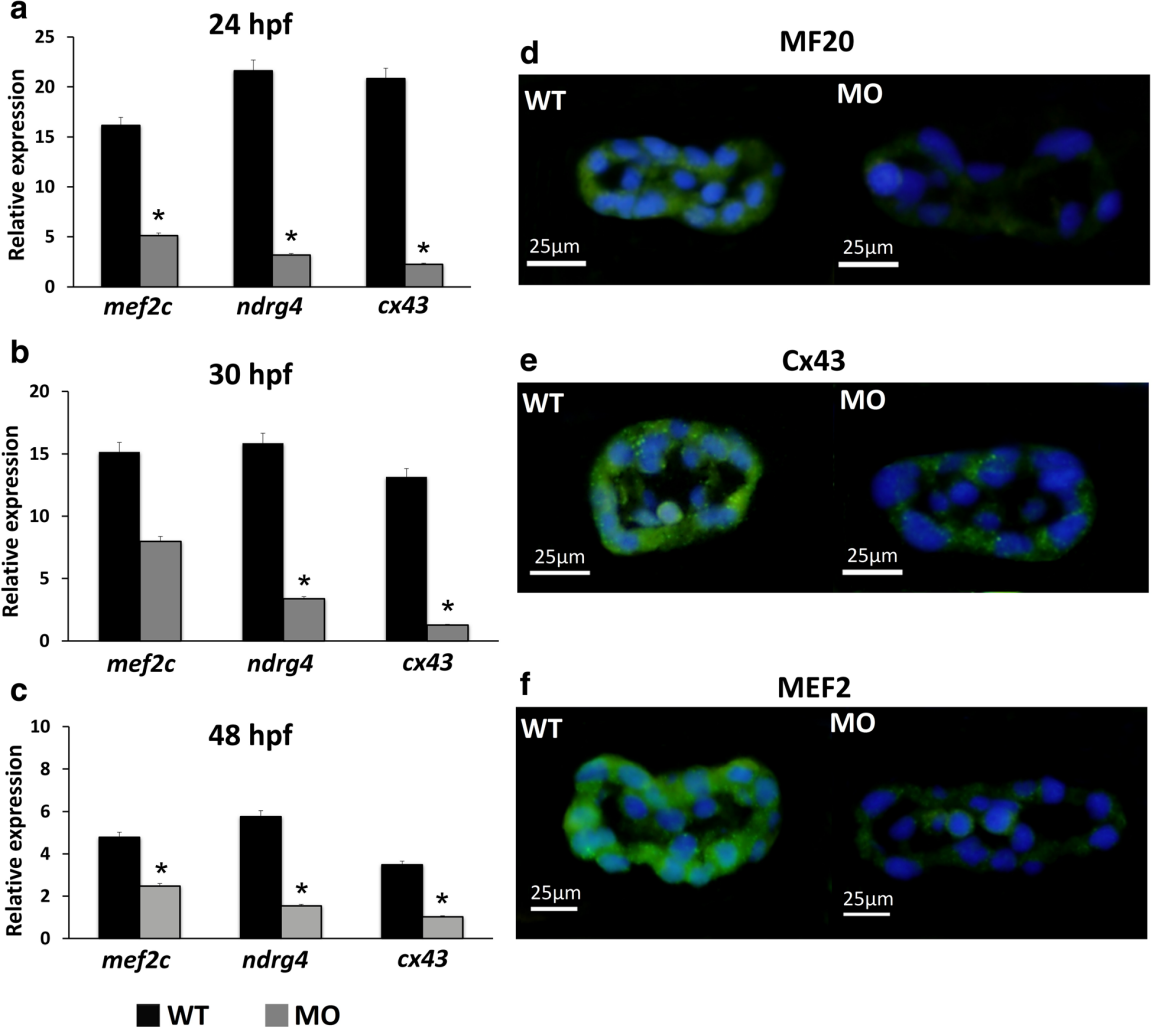
Expression levels of myocardium-related genes and cardiomyocyte proteins in wild-type embryos and *tbx5* morphants. The MO group had significantly decreased expression of myocardium-related genes (*mef2c, ndrg4, cx43*) during the embryonic development stages (24 hpf (**a**), 30 hpf (**b**), and 48 hpf (**c**)) compared with the WT group. Zebrafish embryos at 30 hpf were stained with the myosin heavy chain antibody MF20 (green), connexin-43 antibody Cx43 (green), myocyte enhancer factor-2 antibody MEF2 (green) and counterstained with DAPI (blue) for nucleus identification. The MO group had decreased expression of MF20 (**d**), Cx43 (**e**), and MEF2 (**f**) compared to the WT group. Data are presented as the means ± standard deviations. ^*^P < 0.05 vs. WT. WT, wild-type embryos; MO, *tbx5* morphants. Hpf: hours post-fertilization. The number of specimens was 50, and the number of independent experiments was 3 in each group

To confirm the effects of the *tbx5* morpholino, we observed the phenotypes and their related gene expression in the MO group. By RT-PCR analysis, the MO group had significantly decreased expression levels of myocardium-related genes (*mef2c, ndrg4, cx43*) compared to the WT group during the embryonic development stages of 24 hpf (Fig. 1A), 30 hpf (Fig. 1B), and 48 hpf (Fig. 1C). Compared with the WT group, immunohistochemistry analysis also showed that the MO group had decreased expression levels of cardiomyocyte proteins of MF20 (Fig. 1D), Cx43 (Fig. 1E), and MEF2 (Fig. 1F).

### Expression of human IGF-I-related genes in tbx5 morphants

**Fig. 2:**
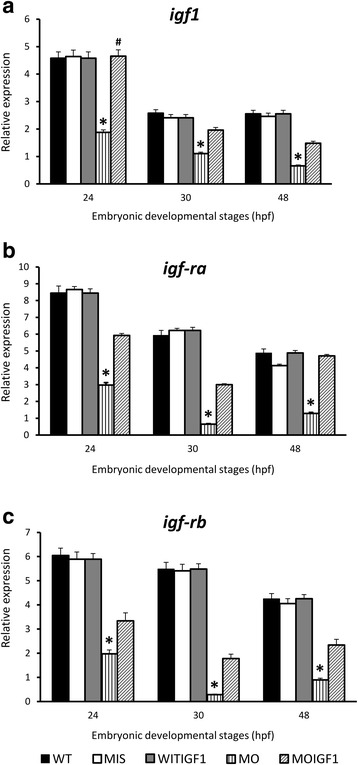
Expression of *igf1, igf1-ra, igf1-rb* in the *tbx5* morphants and IGF-I treated-*tbx5 morphants*. Expression levels of *igf1* (**a**)*, igf1-ra* (**b**) *and igf1-rb* (**c**) were reduced significantly in the MO group throughout all developmental stages. The WTIGF1 and MIS group had similar expression levels as the WT group. Expression of *igf1* in the MOIGF1 group was partially restored and significantly higher than that in the MO group. Data are presented as the means ± standard deviations. ^*^P < 0.05 vs. WT; ^#^*P* < 0.05 MOIGF1 vs. MO. WT: wild-type embryos; MO: *tbx5* morphants; MIS: mismatch *tbx5* morpholino-treated wild-type embryos; WTIGF1: IGF-I-treated wild-type embryos; MOIGF1: IGF-I*-*treated *tbx5* morphants; hpf: hours post-fertilization. The number of specimens was 50, and the number of independent experiments was 3 in each group

As shown in Fig. 2, RT-PCR analysis revealed that the MO group had significantly reduced expression levels of *igf1* (Fig. 2A)*, igf1ra* (Fig. 2B) and *igf1rb* (Fig. 2C) throughout development at 24 hpf, 30 hpf, and 48 hpf. Expression of *igf1* in the MOIGF1 group was partially restored and significantly higher than that in the MO group at 24 hpf. The MIS group and WTIGF1 group had similar expression levels of *igf1, igf1ra,* and *igf1rb* as the WT group throughout the examined developmental stages (Fig. 2).

### Supplementation of human IGF-I partially improves embryonic defects in the tbx5 morphants

**Fig. 3:**
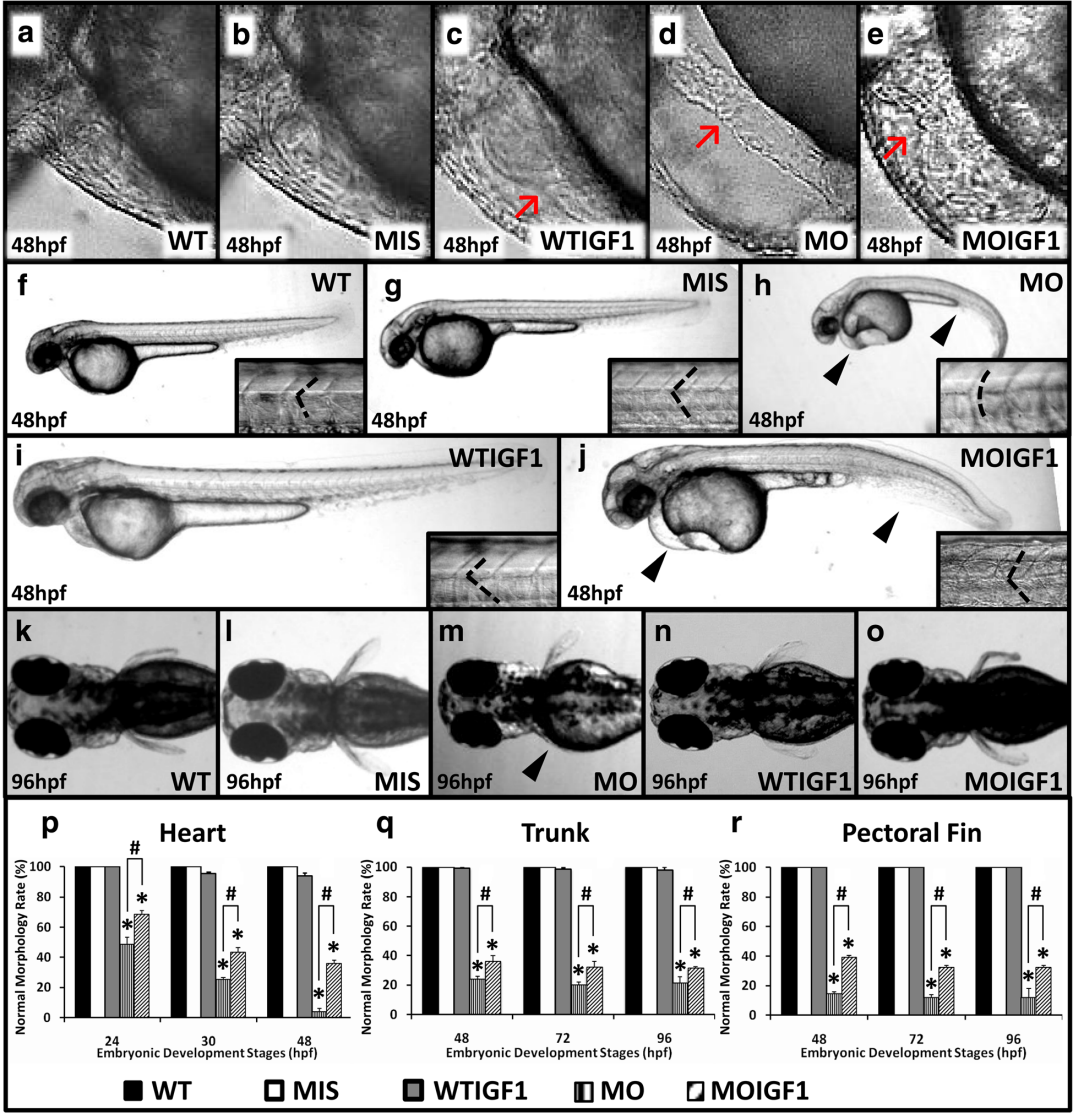
Phenotypes of the *tbx5* morphants and IGF-I-treated *tbx5* morphants. The WT (**a**) and MIS groups (**b**) had normal hearts, and the WTIGF1 (**c**) group had minimal pericardial effusion. The MO group (**d**) had string-like hearts accompanied by a massive pericardial edema, but the appearance of hearts in the MOIGF1 group (**e**) was improved. There were no significant differences observed in the trunks of the WT (**f**), MIS (**g**) and WTIGF1 groups (**i**), in which the trunks were straight and the somites appeared to be “V-shaped”. The trunk of the MO group (**h**) bent severely and showed a “U shape”. The MOIGF1 group (**j**) had partially-restored appearances of the trunks and curvatures of somites. The pectoral fins of the MO group (**m**) were truncated or undeveloped, but the WT (**k**), MIS (**l**), WTIGF1 (**n**), and the MOIGF1 group (**o**) had normal pectoral fins. The MO group had significantly lower normal morphology rates of the hearts (**p**), trunks (**q**) and pectoral fins (**r**). Compared with the MO group, the MOIGF1 group had significantly higher percentages of normal morphology rates of the hearts (**p**), trunks (**q**) and pectoral fins. No defective embryos were found in the WT and MIS group, and almost all of the WTIGF1 group embryos developed well. Red arrow: pericardial effusion; black arrow head: defect site; dashed line: shape of somite border. Data are presented as the means ± standard deviations. ^*^P < 0.05 vs. WT; ^#^P < 0.05 MOIGF1 vs. MO. WT: wild-type embryos; MO: *tbx5* morphants; MIS: mismatch *tbx5* morpholino-treated wild-type embryos; WTIGF1: IGF-I-treated wild-type embryos; MOIGF1: IGF-I*-*treated *tbx5* morphants; hpf: hours post-fertilization. The number of specimens was 50, and the number of independent experiments was 3 in each group

The changes in the cardiac structure of zebrafish embryos were observed via a lateral view. The WT group had an overlapped atrium and ventricle due to cardiac looping formation (Fig. 3A). However, *tbx5* deficiency caused cardiac looping defects in zebrafish embryos and induced characteristic “string-like” heart morphologies, which were frequently accompanied by massive pericardial effusion (Fig. 3D). By contrast, the WT group rarely had pericardial effusion (<1%) (Fig. 3A and B). Minimal pericardial effusion was identified in the IGF-I-treated groups (WTIGF1 and MOIGF1) (Fig. 3C and E). None of the WTIGF1 and MOIGF1 groups had string-like heart phenotypes (Fig. 3P). Thus, the possibility that the string-like heart anomalies were induced by microinjection could be excluded. The MOIGF1 group had a significantly higher percentage of normal cardiac morphology (5% vs. 40% at 48 hpf, *n* = 100 embryos/stage, in triplicate, *P* < 0.05) (Fig. 3P). The normal curvature of somites in the WT group was “V-shaped” (Fig. 3F). Seventy-five percent of the MO group had an abnormal curvature of somites, changing from a sharp “V shape” to a round “U shape” (96 hpf, n = 100, in triplicate) (Fig. 3H, Q). Compared with the MO group, the MOIGF1 group had a significantly higher percentage of normal trunk morphology (20% vs. 30% at 96 hpf, n = 100, in triplicate, P < 0.05) (Fig. 3Q). The anterior pectoral fins of the WT group were bilaterally symmetric and developed normally at 96 hpf (Fig. 3K). The MIS group and WTIGF1 group did not have any abnormalities of the pectoral fins (Fig. 3L, N, R). The MO group had a higher percentage of hypogenesis or complete agenesis of the anterior pectoral fins (Fig. 3M, R). Compared with the MO group, the MOIGF1 group had a significantly higher percentage of normal fin morphology (10% vs. 38% at 96 hpf, n = 100 embryos/stage, in triplicate, P < 0.05) (Fig. 3R).

### Supplementation of human IGF-I normalized expression of phosphorylation-related genes in the IGF-I pathway

**Fig. 4:**
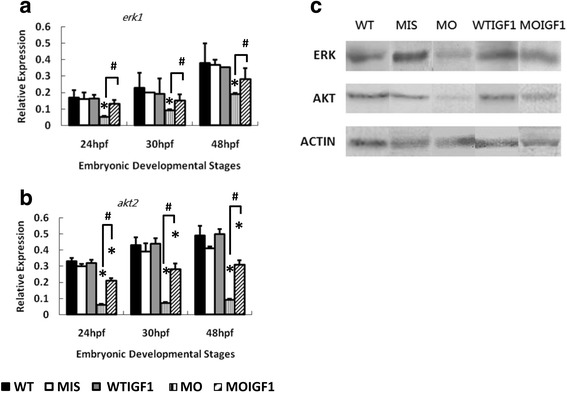
Expression levels of phosphorylation-related genes and proteins in the IGF-I pathway in the *tbx5* morphants and IGF-I-treated *tbx5* morphants. The mRNA expression levels of *erk1* (**a**) and *akt2* (**b**) were reduced significantly in the MO group and restored in the MOIGF1 group. Expression of the ERK1 and AKT2 proteins was significantly reduced in the MO group at 48 hpf but was restored in the MOIGF1 group (**c**). Data are presented as the means ± standard deviations. ^*^P < 0.05 vs. WT; ^#^P < 0.05 MOIGF1 vs. MO. WT: wild-type embryos; MO: *tbx5* morphants; MIS: mismatch *tbx5* morpholino-treated wild-type embryos; WTIGF1: IGF-I-treated wild-type embryos; MOIGF1: IGF-I*-*treated *tbx5* morphants; hpf: hours post-fertilization. The number of specimens was 50, and the number of independent experiments was 3 in each group

According to RT-PCR analysis, the MO group had significantly reduced expression levels of *erk1* (Fig. 4A) and *akt2* (Fig. 4B) compared to the WT group throughout the developmental stages. The expression levels of *akt2* and *erk1* in the MOIGF1 group were restored and were significantly higher than those in the MO group. Both the MIS group and WTIGF1 group had similar expression levels of *akt2* and *erk1* as the WT group (Fig. 4A, B). As shown in Fig. 4C, western blot analysis showed that the MO group had significantly reduced ERK1 and AKT2 protein levels at 48 hpf, whereas the MOIGF1 group had partially normalized expression of ERK1 and AKT2. The expression levels of ERK1 and AKT2 in the MIS group and WTIGF1 group were not different from those in the WT group (Fig. 4C).

### Supplementation of human IGF-I normalized the expression levels of apoptotic genes

**Fig. 5:**
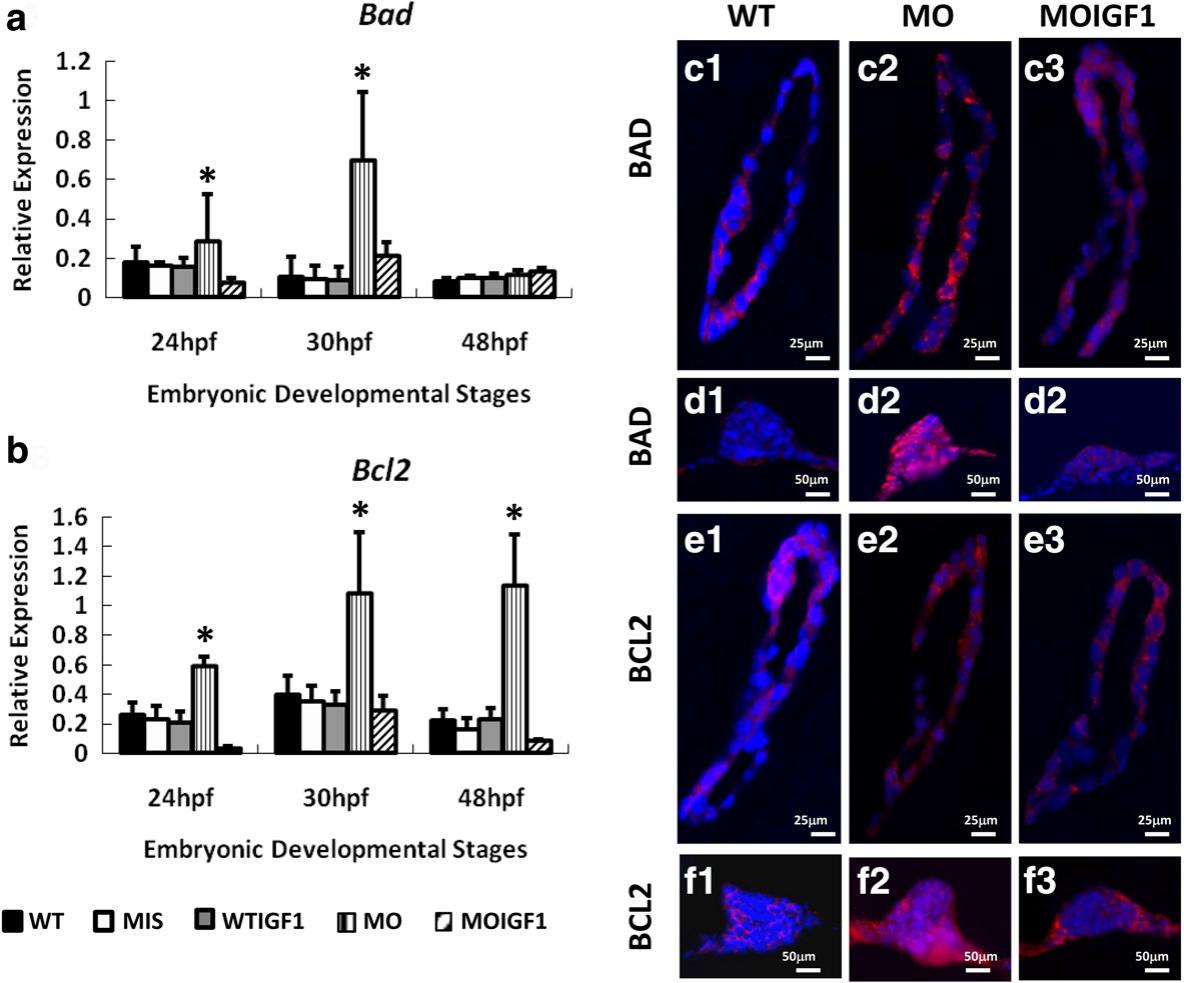
Expression of apoptosis proteins and genes in the *tbx5* morphants and IGF-I-treated *tbx5* morphants. Expression of the *bad* gene was significantly induced in the MO group (**a**) at 24 and 30 hpf, and the expression of *bcl2* was significantly induced in the MO group at 24, 30 and 48 hpf. **b**. There were no significant differences in the expression levels of *bad* and *bcl2* between the WT, WTIGF1 and MOIGF1 groups. Zebrafish embryos at 30 hpf were stained by the proapoptotic-related antibody BAD (red) and anti-apoptotic related antibody BCL2 (red) and were counterstained with DAPI (blue) for nuclear observation. Expression levels in the sagittal sections of hearts are shown in C1–3 and E1–3; expression levels in the transverse sections of pectoral fins are shown in D1–3 and F1–3. The WT group had very low expression of the BAD apoptotic protein (Figure 6C1, 6D1) and BCL2 pro-apoptotic protein (Figure 6E1, 6F1). Expression of the BAD apoptotic protein (Figure 6C2, 6D2) and BCL2 pro-apoptotic protein (Figure 6E2, 6F2) was induced significantly in hearts and pectoral fins in the MO group, whereas expression of BAD (Figure 6C3, 6D3) and BCL2 (Figure 6E3, 6F3) was significantly reduced in the MOIGF1 group. Data are presented as the means ± standard deviations. ^*^P < 0.05 vs WT. WT: wild-type embryos; MO: *tbx5* morphants; MIS: mismatch *tbx5* morpholino-treated wild-type embryos; WTIGF1: IGF-I-treated wild-type embryos; MOIGF1: IGF-I*-*treated *tbx5* morphants; hpf: hours post-fertilization. The number of specimens was 50, and the number of independent experiments was 3 in each group

The expression levels of *bad* and *bcl2* in whole embryos were measured by RT-PCR (Fig. 5A, B). The expression levels of *bad* and *bcl2* in the MO group were elevated significantly at 24 hpf and 30 hpf, but expression of *bad* was within normal limits at 48 hpf (*n* = 50 embryos/stage, in triplicate) (Fig. 5A, B). In the control groups, both the MIS group and WTIGF1 group had similar expression levels of *bad* and *bcl2* as the WT group.

Immunohistochemical analysis at 30 hpf of sagittal sections of hearts is shown in Figure 5C1–3 and 5E1–3. Transverse sections of the pectoral fins are shown in Figure 5D1–3 and 5F1–3. The WT group had very low expression of the BAD apoptotic protein (Figure 5C1, 5D1) and BCL2 pro-apoptotic protein (Figure 5E1, 5F1). The expression levels of the BAD apoptotic protein (Figure 5C2, 5D2) and BCL2 pro-apoptotic protein (Figure 5E2, 5F2) were significantly induced in the hearts and pectoral fins in the MO group, whereas the expression levels of BAD (Figure 5C3, 5D3) and BCL2 (Figure 5E3, 5F3) were significantly reduced in the MOIGF1 group.

**Fig. 6:**
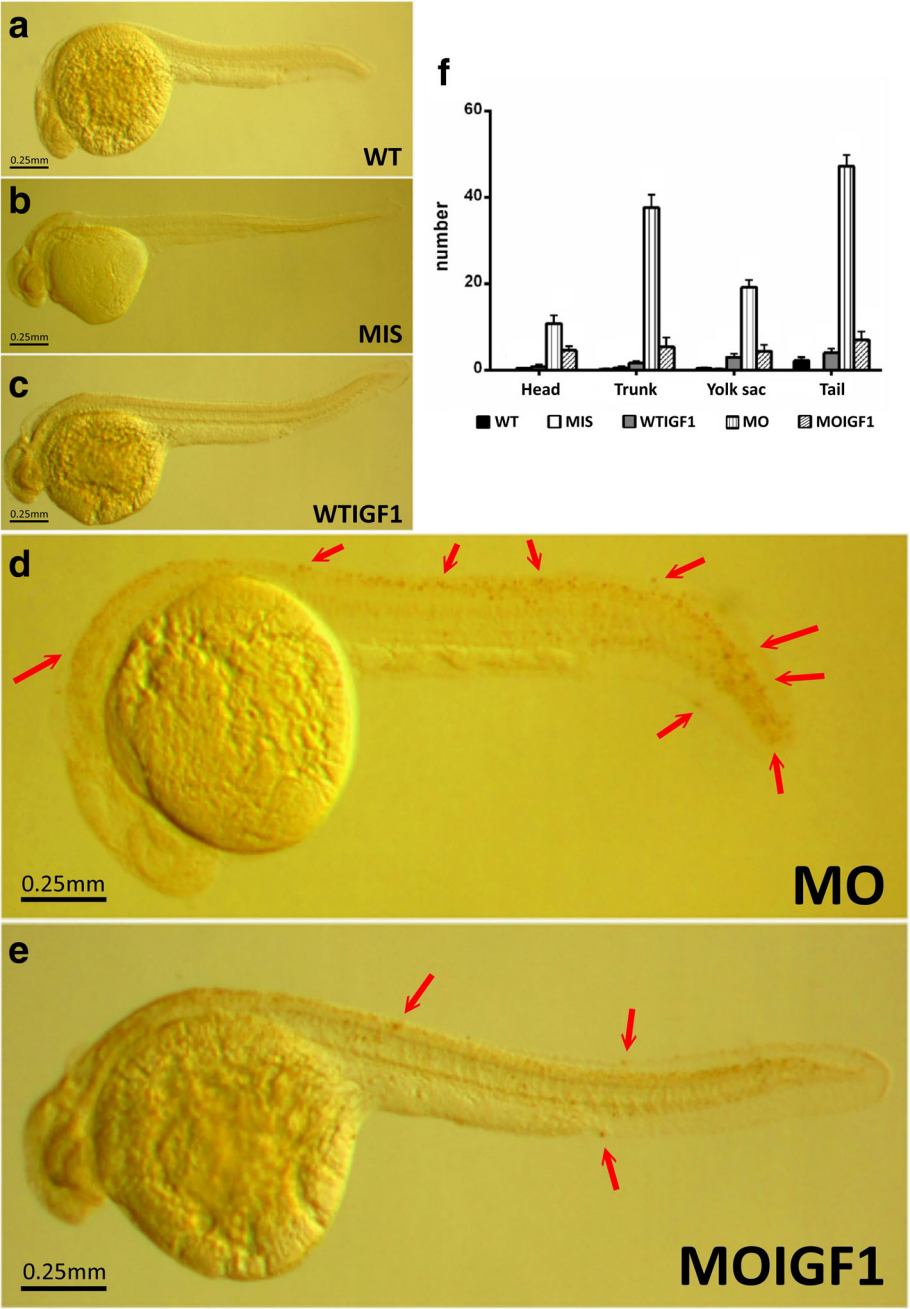
TUNEL assay for the *tbx5* morphants and IGF-I-treated *tbx5* morphants. TUNEL assay revealed no TUNEL-positive cells in the WT (**a**), MIS (**b**), and WTIGF1 (**c**) groups. However, massive numbers of TUNEL-positive cells were visible in the MO group (**d**). In the MOIGF1 group, the amount of TUNEL-positive cells was reduced (**e**). Most of the TUNEL-positive cells were clustered over the tail and trunk, while several were be found over the yolk sac and head (**f**). Arrow, TUNEL-positive cells. Data are presented as the means ± standard deviations. WT: wild-type embryos; MO: *tbx5* morphants; MIS: mismatch *tbx5* morpholino-treated wild-type embryos; WTIGF1: IGF-I-treated wild-type embryos; MOIGF1: IGF-I*-*treated *tbx5* morphants. The number of specimens was 10, and the number of independent experiments was 3 in each group

The TUNEL assay revealed no TUNEL-positive cells in the WT (Fig. 6A), MIS (Fig. 6B), and WTIGF1 (Fig. 6C) groups. However, massive numbers of TUNEL-positive cells were visible in the MO group (Fig. 6D). In the MOIGF1 group, the amount of TUNEL-positive cells was reduced (Fig. 6E). Most of the TUNEL-positive cells were clustered at the tail and trunk, although there were several at the yolk sac and head (Fig. 6F).

**Fig. 7:**
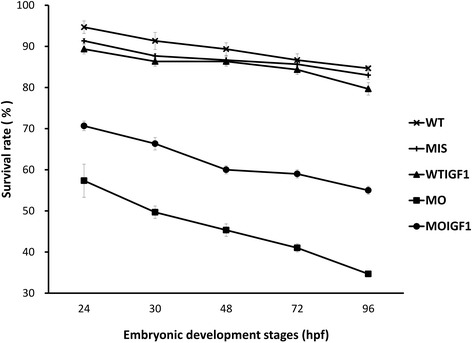
Survival of the *tbx5* morphants and IGF-I-treated *tbx5* morphants. The embryos were examined longitudinally under a microscope for up to 96 hpf. The survival rate of the WT group at 96 hpf was approximately 90%. The MIS and WTIGF1 groups had similar survival rates as the WT group. The MO group had the lowest survival rate, and the survival rate of the MOIGF1 group was significantly improved. WT: wild-type embryos; MO: *tbx5* morphants; MIS: mismatch *tbx5* morpholino-treated wild-type embryos; WTIGF1: IGF-I-treated wild-type embryos; MOIGF1: IGF-I- treated *tbx5* morphants; hpf: hours post-fertilization. The number of specimens was 50, and the number of independent experiments was 3 in each group

Figure 7 shows the results of survival analysis. The MO group had the lowest survival rate (35% at 96 hpf), whereas the survival of the MOIGF1 group was significantly improved (65% at 96 hpf). Both the MIS group and WTIGF group had similar survival rates as the WT group (Fig. 7).

## Discussion

*tbx5* is a regulator of apoptosis and cell growth, and *tbx5* deficiency can induce cell apoptosis and alter cell cycling [22–24]. Although the role of IGF-I is not well-characterized in zebrafish embryogenesis, previous studies have found that *tbx5* morphants have down-regulated *gh* and GH-related genes, including *igf1* [21]. In this study, we found that exogenous recombinant human IGF-I administration ameliorated dysmorphogenesis, increased *akt2* and *erk1* expression*,* normalized *bcl2* and *bad* expression, suppressed cell apoptosis, and improved survival rates in *tbx5* morphants. These results suggest an anti-apoptotic protective effect of IGF-I in zebrafish embryos with *tbx5* deficiency.

Maternal GH and local IGF-I, which are involved in the regulation of embryonic and fetal development in an autocrine or paracrine manner, are produced in extrapituitary tissues and are present before their formation in the pituitary gland. Though both IGF-I and GH are related to embryonic growth and development, they are thought to play different roles in embryogenesis. This study showed that exogenous IGF-I induced positive effects on embryogenesis in wild-type zebrafish embryos (Fig. 3C and I), whereas a previous study reported that exogenous GH had little effect [21]. Additionally, it has been reported that the embryogenic effect of GH probably occurs via an IGF-I-independent mechanism [25]. This phenomenon is consistent with the observation that IGF-dependent growth in prenatal life appears to be largely independent of GH [26]. In postnatal mature individuals, particularly at puberty, IGF-I is produced by the liver and extrahepatic tissues and is related to GH [27]. However, IGF-I plays an independent role in growth, differentiation, regeneration, and metabolism during embryogenesis of vertebrates [28]. Though IGF-I has similar effects as GH in mature individuals, IGF-I can be considered to mediate “insulin-like” and mitogenic effects in cells and embryos.

The in vivo effects of recombinant human and fish IGF-I are mostly conserved throughout vertebrate evolution; however, there are functional differences between mammalian and teleostean IGF, particularly in the interactions and affinities with the different IGF-binding proteins in various species [29]. This study found that exogenous human IGF-I provoked the AKT signaling pathway and subsequent phosphorylation cascades in wild-type zebrafish embryos. Delivery of IGF-I into wild-type zebrafish embryos did not increase the mortality and incidence of embryonic anomalies and also did not affect the expression levels of genes related to phosphorylation, apoptosis, cell cycling, and even cardiomyogenesis. Additionally, supplementation of recombinant human IGF-I in tbx5 morphants ameliorated mortality, restored dysmorphogenesis, induced expression of *igf1* at the mRNA and protein levels as well as expression of *erk1* and *akt2*, provoked the IGF signaling pathway, and activated the phosphorylation pathway, which is highly conserved across species and plays a central role in organogenesis, including in cellular differentiation, cell proliferation, apoptosis, and somatic growth [30]. It is well-known that IGF binds to insulin receptors, activates the PI3K signaling pathway, and subsequently activates the AKT and ERK pathways. The AKT family of serine-threonine kinases, including *akt1*, *akt2,* and *akt3*, has been demonstrated to be involved in promoting cell survival, glucose metabolism, and cellular protein synthesis [31, 32]. Although it remains unclear how human IGF-I functions in zebrafish, our results suggested that the in vivo effects of recombinant human IGF-I were preserved in zebrafish embryos. IGF-I from higher vertebrates still functions in lower vertebrates such as zebrafish. Further investigation is warranted to characterize the complete cDNA sequences and gene structures of zebrafish *igfra* and *igfrb* as well as to identify the origins of both transcripts from human and zebrafish, similar to a recent study for maternal GH and embryonic receptors [33].

The functions of *tbx5* involve many biological processes, such as embryonic limb morphogenesis, heart development, and lung development. Most biological processes can be influenced by IGF-I. Our microarray analysis found that IGF-I up-regulated or down-regulated expression of many RNAs (Table 1). In this study, we only evaluated the effects of IGF-1 on these representative RNAs quantitatively, as well as proteins related to apoptosis (BAD, BCL2 and DAPI) and cardiomyogenesis (MF20, Cx43 and MEF2), and we did not further evaluate the rescue effect of IGF-1 on the other RNAs listed in Table 1. This issue deserves further investigation in future studies. IGF-I can influence most of the *tbx5*-related biological processes in cardiac looping formation (e.g., pattern specific processes, cardiac muscle cell differentiation, cell-cell signaling, negative regulation of the epithelial to mesenchymal transition, positive regulation of cell proliferation). However, exogenous IGF-I only partially rescued these processes and did not completely restore their function. This study found that the MO group had massive pericardial effusion (Fig. 3C). Although the MOIGF1 group (Fig. 3E) had only minimal pericardial effusion, the phenotype was still more abnormal than that of the WT group (Fig. 3A). Similar results were also observed in the phenotypes of the trunk, length of trunk, curvature of the tail, and shape of somites. The MO group had shorter trunk lengths (Fig. 3H) than the WT group (Fig. 3F), and the MOIGF1 (Fig. 3J) had longer trunk lengths than the MO group. Additionally, both the MOIGF1 (Fig. 3J) and WTIGF1 groups (Fig. 3I) had longer trunk lengths than the WT group (Fig. 3F). Therefore, exogenous IGF-I rescued the effects of *tbx5* deficiency regarding the length of the trunk and also stimulated the growth of the embryonic trunk. Although the MOIGF1 group (Fig. 3J) had straighter tails than the MO group (Fig. 3H), the tail was not as straight as that of the WT group (Fig. 3F). Exogenous IGF-I only partially rescued the effect of *tbx5* deficiency regarding the curvature of the tail. The normal shape of somites was a “V shape” (Fig. 3F), and the MO group had “U-shaped” somites (Fig. 3H). As both the MOIGF1 (Fig. 3J) and WTIGF1 groups (Fig. 3I) had normal “V-shaped” somites, exogenous IGF-I rescued the effect of *tbx5* deficiency regarding the shape of somites. Regarding the biological processes that could not be rescued by IGF-I, we listed these genes in Table 1. Moreover, this study only evaluated the effects of IGF-I on expression of RNA in *tbx5* morphants. Whether the function of *tbx5* on DNA (e.g., DNA binding) and RNA (e.g., transcriptional activator activity) could be rescued by IGF-I was beyond the scope of this study. As shown in Table 1, the results of microarray analysis revealed that IGF-1 may also act on several biological processes, including the cell cycle, phosphorylation, cardiac myogenesis, and cardiac morphogenesis, in addition to reducing cell death.

One study of the immunoreactivity of IGF-I in fishes reported that the distribution of IGF-I-IR in this species was tissue-specific and age-dependent [34, 35]. The IGF-I tissue-specific distribution, in which the *igf1* expression level and IGF-I pathway were down-regulated in *tbx5* morphants, was coincident with “premature stops” or “developmental delay” at specific sites (i.e., heart, trunk, and pectoral fins) caused by *tbx5* deficiency in the proper time window, which are also the counterparts of specific areas with aberrant apoptosis and decreased cell cycling [11].

Several reports have suggested that IGF acts as a survival factor for early-stage human follicles, activating the mitotic cell cycle of both the MAPK and PI3 kinase-signaling pathways and regulating cell cycle progression in progenitor cells by multiple growth factors [36–38]. Supplementation of IGF-I to culture media has a facilitating effect on in vitro embryo development, and preimplantation embryos and blastocysts cultured in vitro via decreased apoptosis and increased cell proliferation in mice, rabbits, bovines and humans [22–24, 39]. Our study revealed a similar survival effect of IGF-I on defective zebrafish embryos. The findings that exogenous IGF-I significantly improved the survival of *tbx5* morphants provided further evidence of the effects of exogenous human IGF-I administration.

Currently, anti-sense morpholino administration results in *tbx5* deficiency, but cannot completely abrogate TBX5. Therefore, we were unable to determine whether the positive effects of IGF-I on zebrafish embryos with *tbx5* deficiency depends on the level of TBX5 decrease or on the residual TBX5 function. Though the TBX5 levels increased in the MOIGF1 group compared to the MO group (data not shown), this result is still insufficient to definitively conclude that IGF-I is capable of rescuing anomalies caused by specific gene defects.

## Conclusion

This study found that exogenous recombinant human IGF-I ameliorated dysmorphogenesis, activated the IGF-I pathway, normalized apoptotic gene expression, suppressed cell apoptosis, and improved the survival rate in *tbx5* morphants. The results suggested the potential anti-apoptotic effects of human IGF-I in zebrafish embryos with *tbx5* deficiency.

## Abbreviations

GH: Growth hormone
Hpf: Hour-post-fertilization
IGF-I: Insulin-like growth factors 1
RT-PCR: reverse transcriptase polymerase chain reaction
TUNEL: TdT-UTP nick end labelling

## References

[1] Ahn DG, Kourakis MJ, Rohde LA, Silver LM, Ho RK (2002). T-box gene tbx5 is essential for formation of the pectoral limb bud. Nature.

[2] Begemann G, Ingham PW (2000). Developmental regulation of Tbx5 in zebrafish embryogenesis. Mech Dev.

[3] Fan C, Liu M, Wang Q (2003). Functional analysis of TBX5 missense mutations associated with Holt-Oram syndrome. J Biol Chem.

[4] He ML, Chen Y, Peng Y, Jin D, Du D, Wu J, Lu P, Lin MC, Kung HF (2002). Induction of apoptosis and inhibition of cell growth by developmental regulator hTBX5. Biochem Biophys Res Commun.

[5] Ouimette JF, Jolin ML, L'Honore A, Gifuni A, Drouin J (2010). Divergent transcriptional activities determine limb identity. Nat Commun.

[6] Rallis C, Bruneau BG, Del Buono J, Seidman CE, Seidman JG, Nissim S, Tabin CJ, Logan MP (2003). Tbx5 is required for forelimb bud formation and continued outgrowth. Development.

[7] Siccardi AJ, Garris HW, Jones WT, Moseley DB, D'Abramo LR, Watts SA (2009). Growth and survival of zebrafish (Danio Rerio) fed different commercial and laboratory diets. Zebrafish.

[8] Garrity DM, Childs S, Fishman MC (2002). The heartstrings mutation in zebrafish causes heart/fin Tbx5 deficiency syndrome. Development.

[9] Tamura K, Yonei-Tamura S, Izpisua Belmonte JC (1999). Differential expression of Tbx4 and Tbx5 in zebrafish fin buds. Mech Dev.

[10] Boogerd CJ, Dooijes D, Ilgun A, Mathijssen IB, Hordijk R, van de Laar IM, Rump P, Veenstra-Knol HE, Moorman AF, Barnett P (2010). Functional analysis of novel TBX5 T-box mutations associated with Holt-Oram syndrome. Cardiovasc Res.

[11] Lu J, Tsai T, Choo S, Yeh S, Tang R, Yang A, Lee H, Lu J (2011). Induction of apoptosis and inhibition of cell growth by tbx5 knockdown contribute to dysmorphogenesis in zebrafish embryos. J Biomed Sci.

[12] JH L, JK L, Choo SL, Li YC, Yeh HW, Shiue JF, Yeh VC (2008). Cascade effect of cardiac myogenesis gene expression during cardiac looping in tbx5 knockdown zebrafish embryos. J Biomed Sci.

[13] Bower NI, Johnston IA (2010). Transcriptional regulation of the IGF signaling pathway by amino acids and insulin-like growth factors during myogenesis in Atlantic salmon. PLoS One.

[14] Duan C, Ren H, Gao S (2010). Insulin-like growth factors (IGFs), IGF receptors, and IGF-binding proteins: roles in skeletal muscle growth and differentiation. Gen Comp Endocrinol.

[15] Engert JC, Berglund EB, Rosenthal N (1996). Proliferation precedes differentiation in IGF-I-stimulated myogenesis. J Cell Biol.

[16] Wang Y, Nishida S, Sakata T, Elalieh HZ, Chang W, Halloran BP, Doty SB, Bikle DD (2006). Insulin-like growth factor-I is essential for embryonic bone development. Endocrinology.

[17] Ren J, Samson WK, Sowers JR (1999). Insulin-like growth factor I as a cardiac hormone: physiological and pathophysiological implications in heart disease. J Mol Cell Cardiol.

[18] Laustsen PG, Russell SJ, Cui L, Entingh-Pearsall A, Holzenberger M, Liao R, Kahn CR (2007). Essential role of insulin and insulin-like growth factor 1 receptor signaling in cardiac development and function. Mol Cell Biol.

[19] Saetrum Opgaard O, Wang PH (2005). IGF-I is a matter of heart. Growth Hormon IGF Res.

[20] Schlueter PJ, Royer T, Farah MH, Laser B, Chan SJ, Steiner DF, Duan C (2006). Gene duplication and functional divergence of the zebrafish insulin-like growth factor 1 receptors. FASEB J.

[21] Tsai TC, JK L, Choo SL, Yeh SY, Tang RB, Lee HY, JH L (2012). The paracrine effect of exogenous growth hormone alleviates dysmorphogenesis caused by tbx5 deficiency in zebrafish (Danio Rerio) embryos. J Biomed Sci.

[22] Lin TC, Yen JM, Gong KB, Hsu TT, Chen LR (2003). IGF-1/IGFBP-1 increases blastocyst formation and total blastocyst cell number in mouse embryo culture and facilitates the establishment of a stem-cell line. BMC Cell Biol.

[23] Markham KE, Kaye PL (2003). Growth hormone, insulin-like growth factor I and cell proliferation in the mouse blastocyst. Reproduction.

[24] Spanos S, Becker DL, Winston RM, Hardy K (2000). Anti-apoptotic action of insulin-like growth factor-I during human preimplantation embryo development. Biol Reprod.

[25] Moreira F, Paula-Lopes FF, Hansen PJ, Badinga L, Thatcher WW (2002). Effects of growth hormone and insulin-like growth factor-I on development of in vitro derived bovine embryos. Theriogenology.

[26] Kiess W, Kratzsch J, Keller E, Schneider A, Raile K, Klammt J, Seidel B, Garten A, Schmidt H, Pfaffle R (2005). Clinical examples of disturbed IGF signaling: intrauterine and postnatal growth retardation due to mutations of the insulin-like growth factor I receptor (IGF-IR) gene. Rev Endocr Metab Disord.

[27] Zapf J, Schmid C, Froesch ER (1984). Biological and immunological properties of insulin-like growth factors (IGF) I and II. Clin Endocrinol Metab.

[28] Chen MJ, Kuo YH, Tian XC, Chen TT (2002). Novel biological activities of the fish pro-IGF-I E-peptides: studies on effects of fish pro-IGF-I E-peptide on morphological change, anchorage-dependent cell division, and invasiveness in tumor cells. Gen Comp Endocrinol.

[29] Upton Z, Yandell CA, Degger BG, Chan SJ, Moriyama S, Francis GL, Ballard FJ (1998). Evolution of insulin-like growth factor-I (IGF-I) action: in vitro characterization of vertebrate IGF-I proteins. Comp Biochem Physiol B Biochem Mol Biol.

[30] Laviola L, Natalicchio A, Giorgino F (2007). The IGF-I signaling pathway. Curr Pharm Des.

[31] Amaral IP, Johnston IA (2011). Insulin-like growth factor (IGF) signalling and genome-wide transcriptional regulation in fast muscle of zebrafish following a single-satiating meal. J Exp Biol.

[32] Ren B, Deng Y, Mukhopadhyay A, Lanahan AA, Zhuang ZW, Moodie KL, Mulligan-Kehoe MJ, Byzova TV, Peterson RT, Simons M (2010). ERK1/2-Akt1 crosstalk regulates arteriogenesis in mice and zebrafish. J Clin Invest.

[33] Di Prinzio CM, Botta PE, Barriga EH, Rios EA, Reyes AE, Arranz SE (2010). Growth hormone receptors in zebrafish (Danio Rerio): adult and embryonic expression patterns. Gene Expr Patterns.

[34] Eivers E, McCarthy K, Glynn C, Nolan CM, Byrnes L (2004). Insulin-like growth factor (IGF) signalling is required for early dorso-anterior development of the zebrafish embryo. Int J Dev Biol.

[35] Richardson NA, Anderson AJ, Rimmer MA, Sara VR (1995). Localization of insulin-like growth factor-I immunoreactivity in larval and juvenile barramundi (Lates Calcarifer**)**. Gen Comp Endocrinol.

[36] Frederick TJ, Wood TL (2004). IGF-I and FGF-2 coordinately enhance cyclin D1 and cyclin E-cdk2 association and activity to promote G1 progression in oligodendrocyte progenitor cells. Mol Cell Neurosci.

[37] Louhio H, Hovatta O, Sjoberg J, Tuuri T (2000). The effects of insulin, and insulin-like growth factors I and II on human ovarian follicles in long-term culture. Mol Hum Reprod.

[38] Pozios KC, Ding J, Degger B, Upton Z, Duan C (2001). IGFs stimulate zebrafish cell proliferation by activating MAP kinase and PI3-kinase-signaling pathways. Am J Physiol Regul Integr Comp Physiol.

[39] Makarevich AV, Markkula M (2002). Apoptosis and cell proliferation potential of bovine embryos stimulated with insulin-like growth factor I during in vitro maturation and culture. Biol Reprod.

